# Semi-Synthesis of Different Pyranoflavonoid Backbones and the Neurogenic Potential

**DOI:** 10.3390/molecules28104023

**Published:** 2023-05-11

**Authors:** Corinna Urmann, Lara Bieler, Michael Hackl, Olivia Chia-Leeson, Sebastien Couillard-Despres, Herbert Riepl

**Affiliations:** 1 Organic-Analytical Chemistry, Weihenstephan-Triesdorf University of Applied Sciences, 94315 Straubing, Germany; 2 TUM Campus Straubing for Biotechnology and Sustainability, Technical University of Munich, 94315 Straubing, Germany; 3 Institute of Experimental Neuroregeneration, Spinal Cord Injury and Tissue Regeneration Center Salzburg, Paracelsus Medical University Salzburg, 5020 Salzburg, Austria; 4 Austrian Cluster for Tissue Regeneration, 1200 Vienna, Austria

**Keywords:** xanthohumol, xanthohumol C, pyranoflavonoids, DCX, neurogenic potential, flavanol, flavanone, flavone, aurone

## Abstract

Flavonoids and chalcones are known for their manifold biological activities, of which many affect the central nervous system. Pyranochalcones were recently shown to have a great neurogenic potential, which is partly due to a specific structural motif-the pyran ring. Accordingly, we questioned if other flavonoid backbones with a pyran ring as structural moiety would also show neurogenic potential. Different semi-synthetic approaches starting with the prenylated chalcone xanthohumol, isolated from hops, led to pyranoflavanoids with different backbones. We identified the chalcone backbone as the most active backbone with pyran ring using a reporter gene assay based on the promoter activity of doublecortin, an early neuronal marker. Pyranochalcones therefore appear to be promising compounds for further development as a treatment strategy for neurodegenerative diseases.

## 1. Introduction

Flavonoids are natural products with variable phenolic structures. With over 8000 individual components known, flavonoids can be isolated from a wide range of plants. All flavonoids have in common the basic structure of flavan, however, due to the oxidation and substitution pattern of the C-ring, structural differences may arise [1]. Depending on the degree of oxidation, flavonoids can be divided into different subclasses: flavones (e.g., apigenin, luteolin), flavonols (e.g., quercetin, kaempferol), flavanones (naringenin, hesperetin) and aurones (sulfuretin, maritimetin) [1]. Chalcones are a structural exception, since the C3 bridge is not closed to form a ring, but it is present as an unsaturated carbonyl bond [2,3].

In the central nervous system, flavonoids were shown to dampen microglial activation, to modulate inflammatory processes [4], to possess potent anti-amyloidogenic [5] and antidepressant effects [6,7]. Furthermore, flavonoids improve memory and learning ability [4,8], act neuroprotective [9] and inhibit acetylcholinesterase [10]. Moreover, flavonoids induce neurogenesis, promote neuronal differentiation in pluripotent stem cells and neural progenitors, as well as neurite outgrowth and nerve regeneration [11,12]. All of these are important features to prevent and to reduce the progression of age-related neurodegeneration and justify the growing interest on flavonoids as bioactive compounds.

**Scheme 1:**
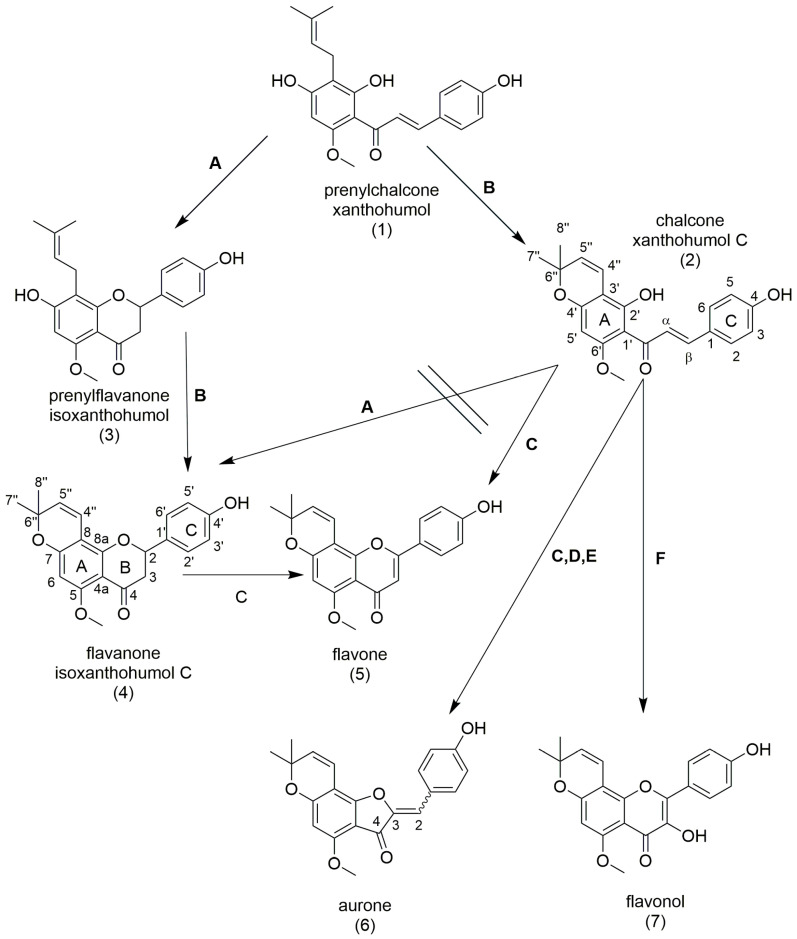
Semi-synthetic synthesis routes to different flavonoid backbones. (**A**) KOH, 0 °C (**B**) DDQ, 1,4-dioxane, MW (**C**) 1,4-dioxane, DDQ (**D**) Hg(OAc)_2_, pyridine (**E**) AgOAc, pyridine (**F**) KOH, H_2_O_2_.

Recently, we identified the chalcone xanthohumol C, found in hops, as a potent inducer of neuronal differentiation. Moreover, it is neuroprotective and supports neuroregeneration [13,14,15]. Compounds showing this activity profile, particularly the induction of neuronal differentiation, are attractive for regenerative medical approaches based on multipotent neural stem cells (NSCs). NSCs are present in the adult human brain throughout life [16] and are capable of self-renewal and differentiation into cell types of the central nervous system such as neurons. NSCs respond to external stimuli such as physical activity [17] or learning [18], but can also respond to small molecules [19]. Since small molecules can be engineered for favorable factors such as bioavailability, they have the potential to induce the body’s own regenerative mechanisms and support healing after ischemic insults and other neurotoxic events. Xanthohumol C belongs to the special flavonoid subclass prenylflavonoids/chalcones, more precisely pyranoflavonoids/chalcones. Although the target(s) is still unknown, the pyran ring seems to be one important structural feature in inducing neuronal differentiation [13]. Therefore, we hypothesized that other flavonoid classes including a pyran ring, beyond chalcones, can also induce neuronal differentiation. Full synthesis of flavonoid scaffolds is challenging and expensive, particularly when the attachment of apolar groups such as prenyl groups or pyran rings is required. Accordingly, the aim of this study was to synthesize different flavonoid scaffolds via a semi-synthetic approach (Scheme 1) and to address their neurogenic potential using a doublecortin (DCX) promoter-based reporter assay.

## 2. Results and Discussion

Xanthohumol (**1**), a prenylated chalcone, is a byproduct of the hop industry, available at a large scale and therefore an ideal educt for further synthesis of prenylated chalcones and flavonoids [20]. Since the prenyl group of xanthohumol (**1**) can be closed to a pyran ring resulting in the regenerative pyranochalcone (**2**) [13], it is also suitable for synthesis of further pyranochalcones and flavonoids. Accordingly, starting with the chalcone xanthohumol (**1**), four different flavonoid backbones with pyran ring and the pyranochalcone (**2**) were synthesized via semi-synthetic routes (Scheme 1).

### 2.1. Synthesis

Xanthohumol C (**2**) was synthesized from xanthohumol (**1**) via semi-synthesis using 2,3-dichlor-5,6-dicyano-1,4-benzochinon (DDQ) [21] but the conventional method was converted to a microwave-assisted method (method B) [15]. Microwave-assisted heating under controlled conditions, such as using a synthesis microwave, is an excellent synthesis technology because it often dramatically reduces reaction times [22]. This was also observed in this study, with a significant reduction in synthesis time from 3 h to 2.45 min and an increase in yield from 20–60% to 77% compared to the conventional synthetic approach using a synthesis microwave device. Isoxanthohumol C (**4**) was obtained by base-catalyzed C-ring closure from xanthohumol (**1**) via isoxanthohumol (**3**) (method A), followed by ring closure and aromatization with DDQ (method B). A direct ring closure reaction of the pyranochalcone to flavanone (method A) was not successful. We attribute this to the hydrogen bonding between the hydroxyl group and the ketone, which can be clearly seen in the ^1^H-NMR from the strong lowfield shift of the hydroxyl group hydrogen involved [23].

The flavone (**5**) was obtained via ring closure and aromatization using DDQ [24,25]. The yield was 51%, which is in the range of the previously published results of 28–42% [25] and 52–58% [24]. Comparing the chalcone xanthohumol C (**2**) and the flavanone isoxanthohumol C (**4**) as an educt, revealed an aurone (**6**) as un unexpected byproduct using the chalcone. That aurone synthesis is possible via this synthesis route was also shown by Imafuku et al. [25]. Starting with isoxanthohumol C (**4**), fewer byproducts were detected shown by LC/MS studies. Accordingly, although the latter method contains one more reaction step, it is preferred due to a simplified purification process of the product. The disappearance of the signals at δ 2.62 ppm (3-H, 1H), δ 2.97 ppm (3-H, 1H) and δ 5.55 ppm (2-H, 1H) and the appearance of the signal at δ 6.58 ppm (3-H, 1H) as well as the signal shifts of C-3 to δ 106.03 ppm and C-2 to δ 159.69 ppm in ^13^C-NMR confirm the obtained structure to be a flavone. In addition, the structure was supported by 2D NMR HSQC and HMBC spectra. In the HMBC spectra a correlation of the proton at H-3 with C-4a (δ 108.02 ppm) and with C-2 (δ 159.69 ppm) appeared, confirming the proposed structure. The 1D and 2D NMR spectra are shown in the Supplementary Information. Such pyranoflavones are already identified in different plants [26] and synthesized [27,28], but a substitution pattern with a methoxy group on A-ring and a hydroxyl group on B-ring is not very common.

The existing methods for aurone (**6**) synthesis include the oxidative cyclisation of 2′-hydroxychalcones using manganic acetate (yield 23–59%) [29], thallium(III)nitrate (yield 43–79%) [30] and mercury(II)acetate (yield 28–62% and 48–78%) [31,32]. All oxidative cyclisations are performed at temperatures ranging from 23–110°C and last from 15 min to 3 h. The formation of aurones strongly depends on the electronic effects of the substituents on both the A- and the B-ring [30]. A full synthetic approach deals with cyclization of 2-(1-hydroxy-3-phenylprop-2-ynyl) phenol using silver nitrate. However, high amounts of expensive silver nitrate rules this method out, accordingly the use of silver acetate was investigated [33]. Here, a method is described for oxidative cyclisation of xanthohumol C (**2**) using silver acetate in pyridine resulting in a yield of 39% for the aurone (**6**). Compared to the mercury(II)acetate supported synthesis, the reaction time is longer (48 h instead of 3 h) and the yield is lower (39% instead of 85%). However, the much lower toxicity of silver acetate is the outstanding advantage of this method. The disappearance of the of α–H signal and the shift of the β-H signal to δ 6.65 ppm (2-H, 1H) in ^1^H-NMR as well as the shifts in ^13^C-NMR confirm the success of aurone (**6**) synthesis, however, the stereoisomerism is not determined. Assignment of the signals was further performed using HSQC and HMBC 2D NMR spectra. The correlation of the 2′-H and the 6′-H signal with the signal of C-2 (δ 111.32) as well as the lack of correlation with the signal of C-4a & C-1 confirm the structure suspected. The 1D and 2D NMR spectra are shown in the Supplementary Information. The analytical data are furthermore in accordance with data already published dealing with prenylated aurones [23]. To the best of our knowledge, the synthesized pyranoaurone has not been described in the literature and its biological activity is not yet known.

The flavonol (**7**) was obtained from xanthohumol C (**2**) via oxidation using hydrogen peroxide in alkaline following a method of Patil et al. [34]. Isolation of the product (**7**) was achieved by recrystallization from acetonitrile/water (1/1). The appearance of the signal at δ 8.78 (3-OH, 1H) in ^1^H-NMR as well as the shift of C-2 and C-3 in ^13^C-NMR confirmed the addition of the hydroxyl group at position C-3. Assignment of signals was performed using HSQC and HMBC 2D NMR spectra, which are shown along with the 1D spectra in the Supporting Information. Such chromenoflavonols methylated on the B-ring were previously extracted from Evodia hupehensis [35].

### 2.2. Neurogenic Potential

**Figure 1:**
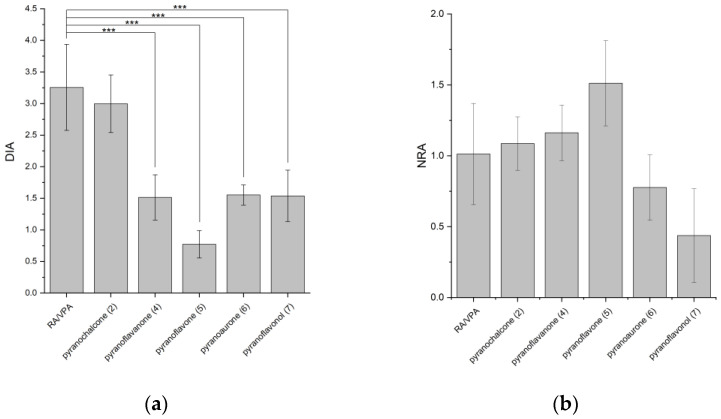
(**a**) Differentiation inducing activity of pyranoflavonoids with different backbones. (c = 10 µM (*n* = 3)) *** *p* < 0.001. (**b**) Normalized *Renilla* activity of pyranoflavonoids with different backbones.(c = 10 µM (*n* = 3)).

The compounds synthesized were investigated for their potential to promote neuronal differentiation using a dual luciferase assay based on the human DCX promoter [36] and primary mouse embryonic forebrain cell cultures. Since stabilization of luciferase can lead to a non-specific increase in signal [37], an interaction assay was performed and no significant influence on luciferase activity was observed at the concentration used. Differentiation-inducing activity (DIA) was calculated from the ratio of the signals originating from the doublecortin promoter-driven firefly luciferase and the constitutive CMV-*Renilla* luciferase. Accordingly, compounds promoting neuronal differentiation have a DIA > 1. Mean DIA values for each compound were calculated from three independent biological replicates. A combination of retinoic acid (RA) (c = 10 µM) and valproic acid (VPA) (c = 50 µM) served as a positive control, in addition to the pyranochalcone (2), for comparison of neurogenic activity (Figure 1). 

Xanthohumol C (**2**), the pyranochalcone, shows a DIA of 2.99 ± 0.45, which does not differ significantly to the DIA of the positive control 3.15 ± 0.68. The closure of the C-ring leading to the flavanone (**4**) reduces the DIA significantly to 1.51 ± 0.30. The flavone (**5**) with a double bond in the C-ring shows a DIA of 0.77 ± 0.21 at a concentration of 10 µM and therefore no differentiation induction. Regarding the flavone structure, the pyran ring seems to have a negative influence on differentiation induction, since apigenin-the natural flavone without a pyran ring-shows a differentiation inducing effect [38,39]. However, flavonol (**7**) with an additional hydroxyl group on the unsaturated C-ring gives a DIA value of 1.54 ± 0.41, which is similar to that of aurone (**6**) (DIA 1.55 ± 0.16) (Figure 1).

*Renilla* activity controlled by the constitutive promoter CMV served as an internal control to assess cell survival and proliferation. Treatment with the positive control (RA/VPA), pyranochalcone (**2**), flavanone (**5**) and aurone (**6**) show similar values of normalized *Renilla* values (NRA), in the range of the control (vehicle) around 1. It seems that there is no strong effect on the proliferation and survival of MEF cells at the concentration of 10 µM used in this study. MEF cells show a trend towards increased proliferation/survival when treated with pyranoflavone (**5**). This is in contrast to flavonol (**7**) treatment, which appears to induce decreased proliferation/viability. In general, the effect of the different flavonoids is strongly dependent on the cell type and the substitution pattern [40].

The most active flavonoid backbone seems to be the chalcone backbone as present in the neurogenic active xanthohumol C (**2**) [15]. An important feature to address the still unknown target seems to be flexibility due to the α,β-unsaturated double bond instead of the C-ring. The chalcone backbone is also known to be beneficial for other bioactivities. For example, the prenylated chalcone backbone also seems to outclass the aurone backbone for the inhibition of cyclooxygenases [23]. The same applies to prenylated chalcones and flavanones as potential cancer chemopreventive agents [41]. 

## 3. Materials and Methods

### 3.1. General Experimental Procedures

NMR spectra were recorded on a JNM-ECS-400 (Jeol, Freising, Germany). Chemical shifts are given in ppm and multiplicity is abbreviated as follows: singlet (s), doublet (d), triplet (t), quartet (q), multiplet (m). Infrared spectra were recorded on a Nicolet 380 FT-IR ATR (Thermo Scientific, Germany). A Shimadzu system (Duisburg, Germany) (2xLC-20AD, SSIL-20AC HT, CTO-20A, SPD-M20) with IT TOF as mass detector and equipped with a column (Phenomenex (Aschaffenburg, Germany), Kinetex C_18_ 2.1 × 50 mm, 2.6u) was used for HRESIMS-analytics using the following method: A (H_2_O + 1% formic acid) and B (MeCN + 1% formic acid); flow rate of 0.4 mL/min. Gradient elution: 00.00–5.35 min 35–95% B; then 5.35–6.35 min 95% B. Thin layer chromatography was executed on TLC silica gel 60 F_254_ alumina sheets (Merck, Taufkirchen, Germany). Compounds were visualized under UV-light at λ = 254 nm, and λ = 360 nm and colored compounds were visualized under daylight. Flash-chromatography was executed on a Puriflash 4250 (Interchim, Mannheim, Germany) with automatic program based on TLC Rf-values. Unless otherwise stated, chemicals for synthesis were purchased from Sigma-Aldrich (Taufkirchen, Germany) and VWR (Damstadt, Germany). Microwave irradiation was carried out with the CEM DiscoverS class single-mode synthesis system (Kampf-Lintfort, Germany) interfaced with a laptop pc running CEM synergy software monitoring the reaction. The temperature was checked by an external infrared sensor in the floor of the cavity. Once the target temperature was reached, the microwave system automatically started to count down the hold time. For reactions CEM Vials 10 mL with snap-on caps were used. The pressure was monitored by a sensor outside the snap-on caps. The upper pressure limit was set to 18 bar. The substances were purified via preparative HPLC before they were used in the in vitro studies and purity can be declared to be >95%. 

### 3.2. Xanthohumol 

((*E*)-1-(2,4-dihydroxy-6-methoxy-3-(3-methylbut-2-en-1-yl)phenyl)-3-(4-hydroxyphenyl)prop-2-en-1-one): Xanthohumol (**1**) was obtained via recrystallization from methanol/H_2_O of the commercial prenylflavonoid-rich Xanthoflav^®^ kindly provided by Hallertauer Hopfenverabeitungsgessllschaft m.b.H. The analytical data are in agreement with the previously published data [42]. Isoxanthohumol was synthesized via basic ring closure [13] and the analytical data are in agreement with the previously published data [13,21,42].

### 3.3. Ring Closure of Prenyl Group to Pyran Ring

To 1 mmol flavonoid/chalcone, 6 mL of 1.4-dioxane and 1 mmol 2,3-dichloro-5,6-dicyano-1,4-benzoquinone (DDQ) were added and heated using microwave to a temperature 70 °C for 2.45 min. The reaction mixture was poured into 30 mL water and was extracted three times with 10 mL EtOAc. The organic layers were combined and washed with saturated NaCl-solution and dried over MgSO_4_. The solvent was removed under reduced pressure and the orange product was purified via flash column chromatography. The analytical data are in agreement with the previously published data [15]. The yield was 77% for chalcone and 51% for flavanone.

Pyranochalcone xanthohumol C (**2**) ((*E*)-1-(5-hydroxy-7-methoxy-2,2-dimethyl-2H-chromen-6-yl)-3-(4-hydroxyphenyl)prop-2-en-1-one): orange solid; ^1^H NMR (*d*_6_-acetone, 400 MHz,) δ 1.43 (s, 6H, 7″-H & 8″-H), 4.00 (s, 3H, OCH_3_), 5.56 (d, 1H, *J* = 10.05 Hz, 5″-H), 6.03 (s, 1H, 5′-H), 6.61 (d, 1H, *J* = 9.87 Hz, 4″-H), 6.91 (d, 2H, *J* = 8.7 Hz, 3-H & 5-H), 7.61 (d, 2H, 2-H & 6-H), 7.75 (m, 1H, β-H), 7.86 (m, 1H, α-H); ^13^C NMR (*d*_6_-acetone, 100 MHz,) δ 28.50 (C-7″ & C-8″), 56.55 (OCH_3_), 78.79 (C-6″), 92.51 (C.5′), 103.33 (C-3′), 106.56 (C-1′), 116.49 (C-3), 116.73 (C-5), 125.13 (C-α), 126.45 (C-5″), 127.95 (C-1), 131.36 (C-2 & C-6), 143.69 (C-β), 160.64 (C-4), 161.00 (C-4′), 162.63 (C-2′), 163.72 (C-6′), 193.48 (CO).

Pyranoflavanone isoxanthohumol C (**4**) (2-(4-hydroxyphenyl)-5-methoxy-8,8-dimethyl-2,3-dihydro-4H,8H-pyrano[2,3-f]chromen-4-one): white solid; ^1^H NMR (*d*_6_-acetone, 400 MHz,) δ 1.41 (d, 6H, 7″-H & 8″-H), 2.62 (dd, 1H, *J* = 16.3, 3.0 Hz, 3-H), 2.97 (dd, 1H, 16,2, 12,8 Hz, 3-H), 3.81 (s, 3H, OCH_3_), 5.41 (d, 1H, *J* = 12.8 Hz, 5″-H), 5.55 (d, 1H, *J* = 10.1 Hz, 2-H), 6.08 (s, 1H, 6-H), 6.53 (d, 1H, *J* = 10.1 Hz, 4″-H), 6.89 (d, 2H, *J* = 8.6 Hz, 3′-H & 5′-H), 7.38 (d, 2H, *J* = 8.5 Hz, 2′-H & 6′-H); ^13^C NMR (*d*_6_-acetone, 100 MHz,) δ 28.22 & 28.53 ((C-7″ & C-8″)), 46.03 (C-2), 56.18 (OCH_3_), 78.39 (C-6″), 79.78 (C-3), 94.37 (C-6), 103.37 (C-8), 106.46 (C-4a), 116.08 (C-4″), 116.67 (C-3′ & C-5′), 127.14 (C-5″), 128.73 (C-2′ & C-6′), 131.11 (C-1′), 158.45 (C-8a), 159.64 (C-4′), 160.26 (C-7), 163.02 (C-5), 188.26 (C-4).

### 3.4. Synthesis of Pyranoflavone

Isoxanthohumol C (**3**) (2 mmol) was dissolved in 10 mL 1,4-dioxane and stirred with 1 mmol DDQ for 3 h at a temperature of 110 °C using conventional oil bath heating. The reaction mixture was poured into 50 mL water and extracted three times with 15 mL EtOAc. The combined organic layers were washed with saturated NaCl-solution and dried over MgSO_4_. The solvent was removed under reduced pressure and the product was purified via flash chromatography. The yield was 51%.

Pyranoflavone (**5**) 2-(4-hydroxyphenyl)-5-methoxy-8,8-dimethyl-4H,8H-pyrano[2,3-f]chromen-4-one: light yellow solid; IR (ATR) ν_max_ 3429, 1643, 1594, 1515, 1490, 1443, 1401, 1344, 1284, 1200, 1178, 1158, 1132; ^1^H NMR (DMSO-*d*_6_, 400 MHz,) δ 10.21 (1H, s, OH); 7.86 (2H, d, *J* = 8.8 Hz, H-2′ & H-6′), 6.89 (3H, t, *J* = 9.6 Hz, H-4″, H-3′ & H-5′), 6.58 (1H, s, H-3), 6.44 (1H, s, 6-H), 5.78 (1H, t. *J* = 10.0 Hz, H-5″), 3.80 (3H, s, OCH_3_), 1.45 (6H, s, H-7″ & H-8″); ^13^C NMR (DMSO-*d*_6_, 100 MHz,) δ 175.61 (C-4), 160.51 (C-4′), 160.00 (C-5), 159.69 (C-2), 157.04 (C-7), 153.03 (C-8a), 128.04 (C-5′), 127.81 (C-2′ & C-6′), 121.38 (C-1′), 115.92 (C-3′ & C-5′), 114.78 (C-4″), 108.02 (C-4a), 106.03 (C-3), 102.5 (C-8), 96.6; (C-6), 77.90 (C-6″), 56.11 (OCH_3_), 27.76 (C-7″ & C-8″) HRESIMS *m/z* 351.1254 (calculated for C_21_H_18_O_5_, 351.1227); TLC (*n*-hexane/EtOAc 1/1) R_F_ 0.05.

### 3.5. Synthesis of Pyranoaurones

Method D (Scheme 1): Xanthohumol C (**2**) (1 mmol) was dissolved in 10 mL pyridine, 1.2 eq mercury(II)acetate was added and the mixture was stirred for 3 h at 110 °C. The reaction mixture was acidified with HCl (3 M), diluted with 100 mL water and extracted for three times with 15 mL EtOAc. The combined organic layers were washed with saturated NaCl-solution and dried over MgSO_4_. The solvent was removed under reduced pressure and the product was purified via flash chromatography. The yield was 85% (0.17 mmol). Method E (Scheme 1): Xanthohumol C (**2**) (1 mmol) was dissolved in 10 mL pyridine and 7 mmol silver acetate was added. The reaction mixture was stirred for 48 h at room temperature in the dark. The silver acetate was removed by filtration and the reaction mixture was acidified with HCl (3 M), 30 mL water was added and it was extracted 3 times with 15 mL EtOAc. The combined organic layers were washed with saturated NaCl-solution and dried over MgSO_4_. The solvent was removed under reduced pressure and the product was purified via flash chromatography. The yield was 39%.

Pyranoaurone (**6**) 2-(4-hydroxybenzylidene)-4-methoxy-7,7-dimethyl-7H-furo[2,3-f]chromen-3(2H)-one: yellow solid; IR (ATR) ν_max_ 3311, 2925, 1687, 1658, 1621, 1581, 1516, 1463, 1440, 1416, 1362, 1343, 1263, 1203, 1176, 1158, 1081, 1011, 953, 899, 880, 855, 838, 811, 756, 730, 701, 667, 638, 618, 586, 550, 527, 495, 480, 454, 440; ^1^H NMR (DMSO-*d*_6_, 400 MHz,) δ 10.08 (1H, s, OH), 7.80 (2H, d, *J* = 8.7 Hz), 6.87 (2H, d, *J* = 8.7 Hz, H-3′ & H-5′), 6.73 (1H, d, *J* = 9.9 Hz, H-4″), 6.65 (1H, s, H-2), 6.27 (1H, s, H-6), 5.79 (1H, d, *J* = 10.1 Hz, H-5″), 3.86 (3H, s, OCH_3_), 1.46 (6H, s, H-7″ & H-8″); ^13^C NMR (DMSO-*d*_6_, 100 MHz,) δ 178.62 (C-4), 162.26 (C-7), 162.15 (C-8a), 159.67 (C-4′), 159.21 (C-5), 146.20 (C-3), 133.23 (C-2′ & C-6′), 127.96 (C-5″), 123.39 (C-1′), 116.08 (C-3′ & C-5′), 110.76 (C-2). 104.72 (C-4a), 98.97 (C-8), 95.03 (C-6), 78.85 (C-6″), 56.22 (OCH_3_), 27.94 (C-7″ & C-8″); HRESIMS *m/z* 351.1251 (calculated for C_21_H_18_O_5_, 351.1227); TLC (*n*-hexane/EtOAc 1/1) R_f_ 0.47.

### 3.6. Synthesis of Flavonols

Xanthohumol C (**2**) (1 mmol) was dissolved in 3 mL potassium hydroxide (8 M) and 750 µL hydrogen peroxide (30%) was added. The mixture was stirred for 2 h at room temperature, acidified with HCl (3 M) and extracted three times with 10 mL EtOAc. The combined organic layers were washed with saturated NaCl-solution and dried over MgSO_4_. The solvent was removed under reduced pressure and the product was purified via flash chromatography und recrystallized from acetonitrile/water 1:1. The yield was 20%.

Pyranoflavonol (**7**) 3-hydroxy-2-(4-hydroxyphenyl)-5-methoxy-8,8-dimethyl-4H,8H-pyrano[2,3-f]chromen-4-one: yellow solid; IR (ATR) ν_max_ 3433, 3214, 2923, 1610, 1575, 1562, 1513, 1448, 1425, 1361, 1334, 1284, 1248, 1220, 1201, 1175, 1153, 1124, 1023, 839, 825, 586; ^1^H NMR (DMSO-*d*_6_, 400 MHz,) δ 9.99 (1H, s, OH-4′), 8.78 (1H, s, OH-3), 8.01 (2H, d, *J* = 8.9 Hz, H-2′ & H-6′), 6.42 (1H, s, H-6), 5.76 (1H, d, *J* = 10 Hz, H-5″), 3.84 (3H, s, OCH_3_), 1.44 (6H, s, H-7″ & H-8″); ^13^C NMR (DMSO-*d*_6_, 100 MHz,) δ 171.05 (C-4), 159.95 (C-5), 128.72 (C-2′ & C-6′), 127.74 (C-5″), 121.95 (C-1′), 115.53 (C-3′ & C-5′), 114.66 (C-4″), 106.07 (C-4′), 101.68 (C-8), 95.95 (C-6), 77.98 (C-6″), 56.25 (OCH_3_), 27.77 (C-7″ & C-8″); HRESIMS *m/z* 367.1204 (calculated for C_21_H_18_O_6_, 367.1176); TLC (*n*-hexane/EtOAc 1/1) R_f_ 0.28.

### 3.7. Reporter Assay

The assay was performed as previously described in Oberbauer et al. [15]. In brief, primary mouse embryonic forebrain (MEF 16.5) cultures, were obtained from NMRI mice (16.5 dpc) (Charles River Laboratories, Sulzfeld, Germany), cultivated in neurobasal (NB) medium (Gibco BRL, Karlsruhe, Germany) supplemented with 2% (*v/v*) B27 (Gibco BRL, Karlsruhe, Germany), 2 mM L-glutamine, 100 U/mL Penicillin, 100 μg/mL Streptomycin, 20 ng/mL Epidermal Growth Factor-(rhEGF), 20 ng/mL Fibroblast Growth Factor (rhFGF) (R&D Systems, Wiesbaden-Nordenstadt, Germany), 2 µg/mL Heparin and maintained in a humidified atmosphere at 37 °C and 5% CO_2_.

Luciferase assays were performed using a non-commercial dual luciferase enzyme assay system [36]. In this system, the MEF cells were co-transfected with a vector encoding the firefly luciferase reporter gene driven by the DCX promoter and a control vector driving *Renilla* luciferase under the CMV promoter to normalize the DCX-driven gene expression. Transfected cells were resuspended in NB, supplemented with 2% (*v/v*) B27, 2 mM L-glutamine, 100 U/mL Penicillin, 100 μg/mL Streptomycin and 1% fetal calf serum (FCS), and seeded into 100 µg/mL poly-ornithine/5 µg/mL laminin coated white 96-well plates (25,000 cells/well). One day after cell seeding, the medium was replaced by fresh NB-B27 with 1% FCS containing 10 µM of the flavonoids to be tested. Each substance was applied for three days in technical tetraplicates. The bioluminescent measurement was performed in a TriStar Multimode Microplate Reader LB 941 (Berthold Technologies, Wien, Austria) according to the manufacturer’s instructions.

### 3.8. Statistics

For one-way ANOVA with Bonferroni test, OriginPro 2021b (Originlab Corporation) was used.

## 4. Conclusions

In this study, different pyranoflavonoids were produced via semi-synthetic approaches and the neurogenic potential of the different flavonoid backbones was investigated. The semi-synthetic yields were improved by microwave irradiation compared to the conventional heating method. Furthermore, the possibility of using silver acetate for aurone synthesis instead of toxic reagents such as thallium or mercury acetate is noteworthy. The various semi-synthetic isomerization reactions are suitable for further structure-activity studies of natural products, as mostly simple one-pot, one-step reactions were used, providing an easily accessible source of different flavonoid backbones. Furthermore, we have shown that the chalcone backbone, in addition to the pyran ring, is beneficial for neurogenic activity according to the DCX promoter reporter assay. Therefore, pyranochalcones appear to be promising compounds for further development as a strategy for the treatment of neurodegenerative diseases.

## Data Availability

Not applicable.

## Supplementary Materials

The following supporting information can be downloaded at: https://www.mdpi.com/article/10.3390/molecules28104023/s1, Figure S1: (**a**) ^1^H-NMR and (**b**) ^13^C-NMR of pyranochalcone (**2**); Figure S2: (**a**) ^1^H-NMR and (**b**) ^13^C-NMR of pyranoflavanone (**4**); Figure S3: (**a**) ^1^H-NMR, (**b**) ^13^C-NMR, (**c**) HSQC and (**d**) HMBC of pyranoflavone (**5**); Figure S4: (**a**) ^1^H-NMR, (**b**) ^13^C-NMR, (**c**) HSQC and (**d**) HMBC of pyranoaurone (**6**); Figure S5: (**a**) ^1^H-NMR, (**b**) ^13^C-NMR, (**c**) HSQC and (**d**) HMBC of pyranoflavonol (**7**).

## References

[1] Kumar S., Pandey A.K. (2013). Chemistry and biological activities of flavonoids: An overview. Sci. World J..

[2] Elias D., Beazely M., Kandepu N. (1999). Bioactivities of chalcones. Curr. Med. Chem..

[3] Jasim H.A., Nahar L., Jasim M.A., Moore S.A., Ritchie K.J., Sarker S.D. (2021). Chalcones: Synthetic Chemistry Follows Where Nature Leads. Biomolecules.

[4] Spencer J.P., Vafeiadou K., Williams R.J., Vauzour D. (2012). Neuroinflammation: Modulation by flavonoids and mechanisms of action. Mol. Asp. Med..

[5] Uddin M.S., Kabir M.T., Niaz K., Jeandet P., Clement C., Mathew B., Rauf A., Rengasamy K.R.R., Sobarzo-Sanchez E., Ashraf G.M. (2020). Molecular Insight into the Therapeutic Promise of Flavonoids against Alzheimer’s Disease. Molecules.

[6] Guan L.P., Liu B.Y. (2016). Antidepressant-like effects and mechanisms of flavonoids and related analogues. Eur. J. Med. Chem..

[7] Hritcu L., Ionita R., Postu P.A., Gupta G.K., Turkez H., Lima T.C., Carvalho C.U.S., de Sousa D.P. (2017). Antidepressant Flavonoids and Their Relationship with Oxidative Stress. Oxid. Med. Cell. Longev..

[8] Kim D.H., Kim S., Jeon S.J., Son K.H., Lee S., Yoon B.H., Cheong J.H., Ko K.H., Ryu J.H. (2009). Tanshinone I enhances learning and memory, and ameliorates memory impairment in mice via the extracellular signal-regulated kinase signalling pathway. Br. J. Pharmacol..

[9] Vauzour D., Vafeiadou K., Rodriguez-Mateos A., Rendeiro C., Spencer J.P. (2008). The neuroprotective potential of flavonoids: A multiplicity of effects. Genes Nutr..

[10] Xie Y., Yang W., Chen X., Xiao J. (2014). Inhibition of flavonoids on acetylcholine esterase: Binding and structure-activity relationship. Food Funct..

[11] Cichon N., Saluk-Bijak J., Gorniak L., Przyslo L., Bijak M. (2020). Flavonoids as a Natural Enhancer of Neuroplasticity-An Overview of the Mechanism of Neurorestorative Action. Antioxidants.

[12] Spencer J.P. (2007). The interactions of flavonoids within neuronal signalling pathways. Genes Nutr..

[13] Urmann C., Bieler L., Priglinger E., Aigner L., Couillard-Despres S., Riepl H.M. (2021). Neuroregenerative Potential of Prenyl- and Pyranochalcones: A Structure-Activity Study. J. Nat. Prod..

[14] Bieler L., Vogl M., Kirchinger M., Urmann C., Riepl H., Bandtlow C., Klimaschewski L., Aigner L., Couillard-Despres S. (2019). The Prenylflavonoid ENDF1 Overrules Central Nervous System Growth Inhibitors and Facilitates Regeneration of DRG Neurons. Front. Cell. Neurosci..

[15] Oberbauer E., Urmann C., Steffenhagen C., Bieler L., Brunner D., Furtner T., Humpel C., Baumer B., Bandtlow C., Couillard-Despres S. (2013). Chroman-like cyclic prenylflavonoids promote neuronal differentiation and neurite outgrowth and are neuroprotective. J. Nutr. Biochem..

[16] Eriksson P.S., Perfilieva E., Bjork-Eriksson T., Alborn A.M., Nordborg C., Peterson D.A., Gage F.H. (1998). Neurogenesis in the adult human hippocampus. Nat. Med..

[17] van Praag H., Schinder A.F., Christie B.R., Toni N., Palmer T.D., Gage F.H. (2002). Functional neurogenesis in the adult hippocampus. Nature.

[18] Gould E., Beylin A., Tanapat P., Reeves A., Shors T.J. (1999). Learning enhances adult neurogenesis in the hippocampal formation. Nat. Neurosci..

[19] Couillard-Despres S., Wuertinger C., Kandasamy M., Caioni M., Stadler K., Aigner R., Bogdahn U., Aigner L. (2009). Ageing abolishes the effects of fluoxetine on neurogenesis. Mol. Psychiatry.

[20] Urmann C., Riepl H. (2020). Semi-Synthetic Approach Leading to 8-Prenylnaringenin and 6-Prenylnaringenin: Optimization of the Microwave-Assisted Demethylation of Xanthohumol Using Design of Experiments. Molecules.

[21] Jain A.C., Gupta R.C., Sarpal P.D. (1978). Synthesis of (+/−) Lupinifolin, Di-O-Methyl Xanthohumol and Isoxanthohumol and Related Compounds. Tetrahedron.

[22] Kappe C.O., Dallinger D. (2006). The impact of microwave synthesis on drug discovery. Nat. Rev..

[23] Tronina T., Strugala P., Poplonski J., Wloch A., Sordon S., Bartmanska A., Huszcza E. (2017). The Influence of Glycosylation of Natural and Synthetic Prenylated Flavonoids on Binding to Human Serum Albumin and Inhibition of Cyclooxygenases COX-1 and COX-2. Molecules.

[24] Venkatesan P., Maruthavanan T. (2011). Synthesis of substituted flavone derivatives as potent antimicrobial agents. Bull. Chem. Soc. Ethiop..

[25] Imafuku K., Honda M., McOmie J.F.W. (1987). ChemInform Abstract: Cyclodehydrogenation of 2′-Hydroxychalcones (I) with DDQ: A Simple Route for Flavones (III) and Aurones (IV). ChemInform.

[26] Banerji A., Luthria D.L., Prabhu B.R. (1988). Prenylated compounds from Atalantia racemosa: Isolation and synthesis of two pyranoflavones. Phytochemistry.

[27] Banerji A., Luthria D.L. (1990). Benzene Induced1H NMR Shifts of Chromeno-Compounds: An Aid to Differentiate Linear and Angular Chromenoflavones. Spectrosc. Lett..

[28] Banerji A., Luthria D.L. (2006). Structural Elucidation of Isomeric Pyranoflavones by 2D-Noesy. Spectrosc. Lett..

[29] Kurosawa K. (1969). Manganic Acetate Oxidation of 2′-Hydroxychalcones. Bull. Chem. Soc. Jpn..

[30] Thakkar K., Cushman M. (1995). A Novel Oxidative Cyclization of 2′-Hydroxychalcones to 4,5-Dialkoxyaurones by Thallium(III) Nitrate. J. Org. Chem..

[31] Sekizaki H. (1988). Synthesis of 2-Benzylidene-3(2H)-benzofuran-3-ones (Aurones) by Oxidation of 2′-Hydroxychalcones with Mercury(II) Acetate. Bull. Chem. Soc. Jpn..

[32] Roussaki M., Costa Lima S., Kypreou A.M., Kefalas P., Cordeiro da Silva A., Detsi A. (2012). Aurones: A promising heterocyclic scaffold for the development of potent antileishmanial agents. Int. J. Med. Chem..

[33] Hwang W., Kim H., Choi H., Kim J., Jeon W.H., Lee P.H., Lee K. (2018). Synthesis of Aurones through Silver-catalyzed Intramolecular Cyclization from o-Alkynonylphenols. Bull. Korean Chem. Soc..

[34] Patil V.C. (2012). Synthesis and in vitro antiplaque activity of chalcone, flavonol and flavanol derivatives. Int. J. Pharm. Sci. Res..

[35] Reisch J., Hussain R., Szendrei K., Adesina S. (1985). Extractives from Evodia hupehensis fruit hull, peduncle, twig and leaf. CIV: Natural product chemistry. Pharmazie.

[36] Karl C., Couillard-Despres S., Prang P., Munding M., Kilb W., Brigadski T., Plotz S., Mages W., Luhmann H., Winkler J. (2005). Neuronal precursor-specific activity of a human doublecortin regulatory sequence. J. Neurochem..

[37] Auld D.S., Lovell S., Thorne N., Lea W.A., Maloney D.J., Shen M., Rai G., Battaile K.P., Thomas C.J., Simeonov A. (2010). Molecular basis for the high-affinity binding and stabilization of firefly luciferase by PTC124. Proc. Natl. Acad. Sci. USA.

[38] Taupin P. (2009). Apigenin and related compounds stimulate adult neurogenesis. Mars, Inc., the Salk Institute for Biological Studies: WO2008147483. Expert Opin. Ther. Pat..

[39] Namsi A., Nury T., Hamdouni H., Yammine A., Vejux A., Vervandier-Fasseur D., Latruffe N., Masmoudi-Kouki O., Lizard G. (2018). Induction of Neuronal Differentiation of Murine N2a Cells by Two Polyphenols Present in the Mediterranean Diet Mimicking Neurotrophins Activities: Resveratrol and Apigenin. Diseases.

[40] Kuntz S., Wenzel U., Daniel H. (1999). Comparative analysis of the effects of flavonoids on proliferation, cytotoxicity, and apoptosis in human colon cancer cell lines. Eur. J. Nutr..

[41] Gerhauser C. (2005). Beer constituents as potential cancer chemopreventive agents. Eur. J. Cancer.

[42] Stevens J.F., Ivancic M., Hsu V.L., Deinzer M.L. (1997). Prenylflavonoids from Humulus lupulus. Phytochemistry.

