# Supplementary material for: Semi-Synthesis of Different Pyranoflavonoid Backbones and the Neurogenic Potential
Source: Molecules. 2023 May 11;28(10):4023. doi: 10.3390/molecules28104023 (PMC10222040; doi:10.3390/molecules28104023)
Supplement: Supplementary file 1 [file molecules-28-04023-s001.zip › molecules-2345833-supplementary.pdf]

Supporting Information

Semi-Synthesis of Different Pyranoflavonoid Backbones and the Neurogenic Potential

Corinna Urmann [1,2,*], Lara Bieler [3,4], Michael Hackl [2], Olivia Chia-Leeson [2], Sebastien Couillard-Despres [3,4] and Herbert Riepl [1,2,*]

[1] Organic-Analytical Chemistry, Weihenstephan-Triesdorf University of Applied Sciences, 94315 Straubing, Germany
[2] TUM Campus Straubing for Biotechnology and Sustainability, Technical University of Munich, 94315 Straubing, Germany
[3] Institute of Experimental Neuroregeneration, Spinal Cord Injury and Tissue Regeneration Center Salzburg, Paracelsus Medical University Salzburg, 5020 Salzburg, Austria
[4] Austrian Cluster for Tissue Regeneration, 1200 Vienna, Austria
* Correspondence: corinna.urmann@tum.de (C.U.); herbert.riepl@hswt.de (H.R.)

Content

1. NMR spectra of pyranochalcone (2) 2
2. NMR spectra of pyranoflavanone (4) 3
3. NMR spectra of pyranoflavone (5) 4
4. NMR spectra of pyranoaurone (6) 6
5. NMR spectra of pyranoflavonol (7) 8

Supporting Information -Semi-Synthesis of Different Pyranoflavonoid Backbones and the Neurogenic Potential

1. NMR spectra of pyranochalcone (2)

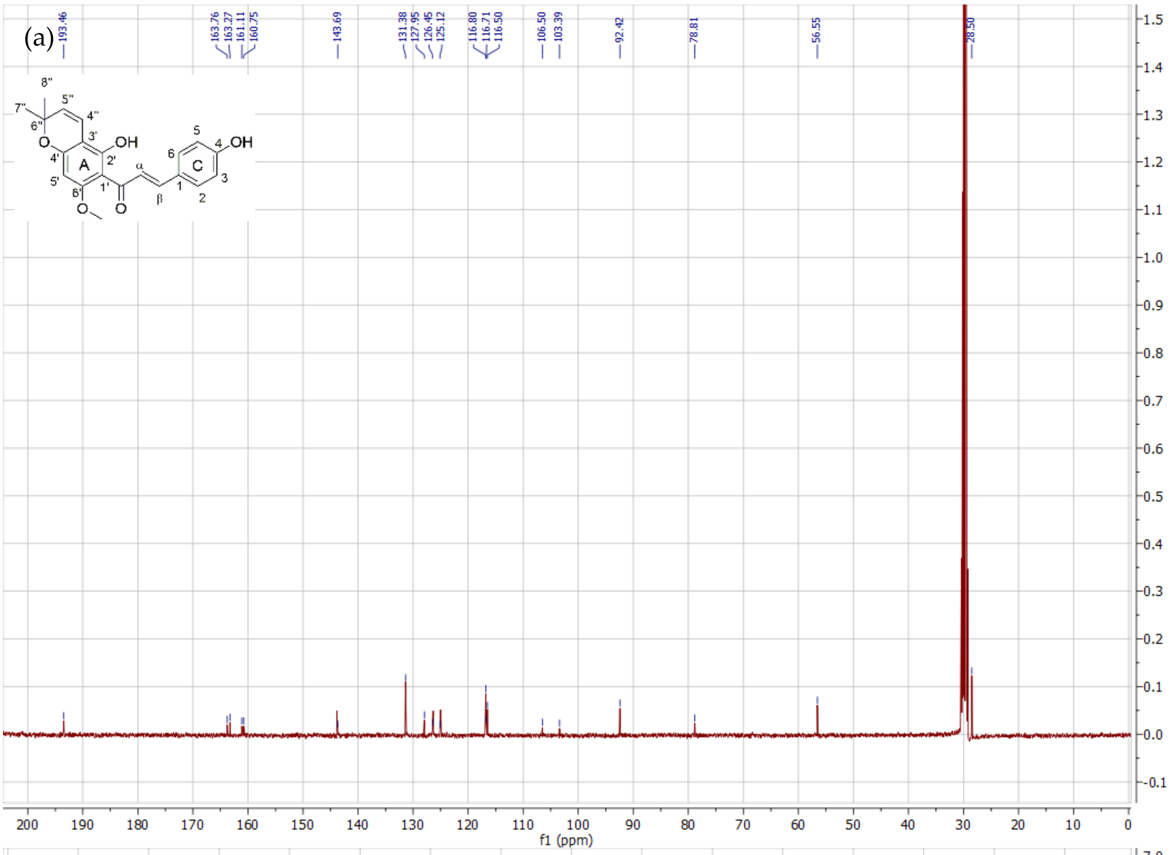

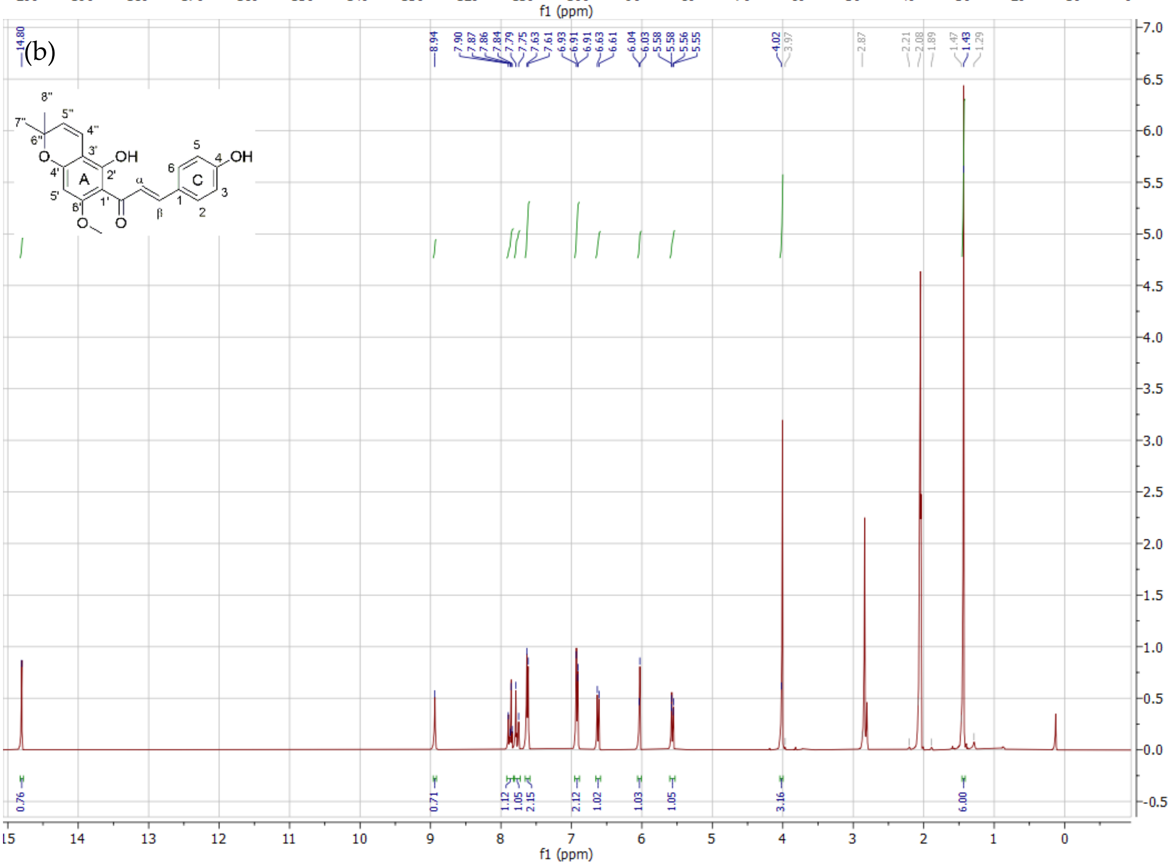

Figure S1. (**a**) ^{1}H-NMR and (**b**) ^{13}C-NMR of pyranochalcone (**2**).

2. NMR spectra of pyranoflavanone (4)

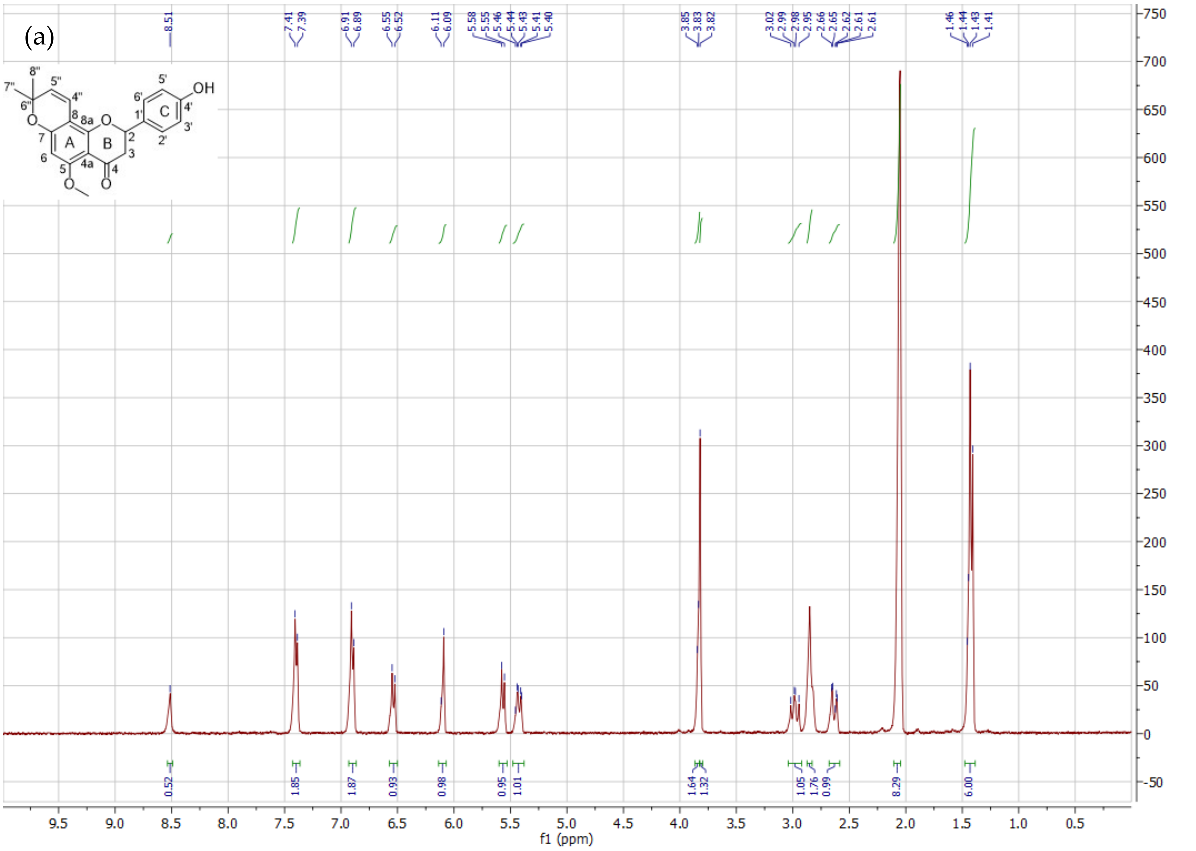

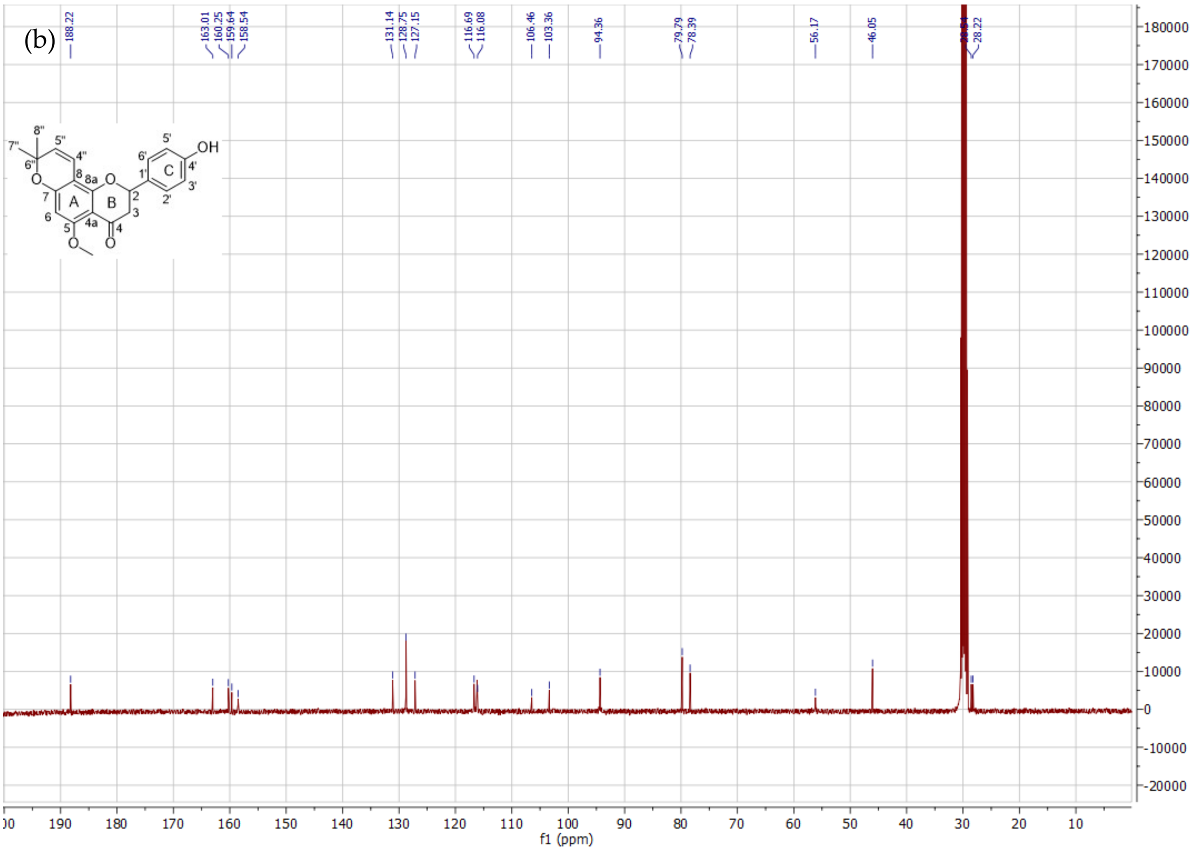

Figure S2. (**a**) ^{1}H-NMR and (**b**) ^{13}C-NMR of pyranoflavanone (**4**).

Supporting Information -Semi-Synthesis of Different Pyranoflavonoid Backbones and the Neurogenic Potential

3. NMR spectra of pyranoflavone (5)

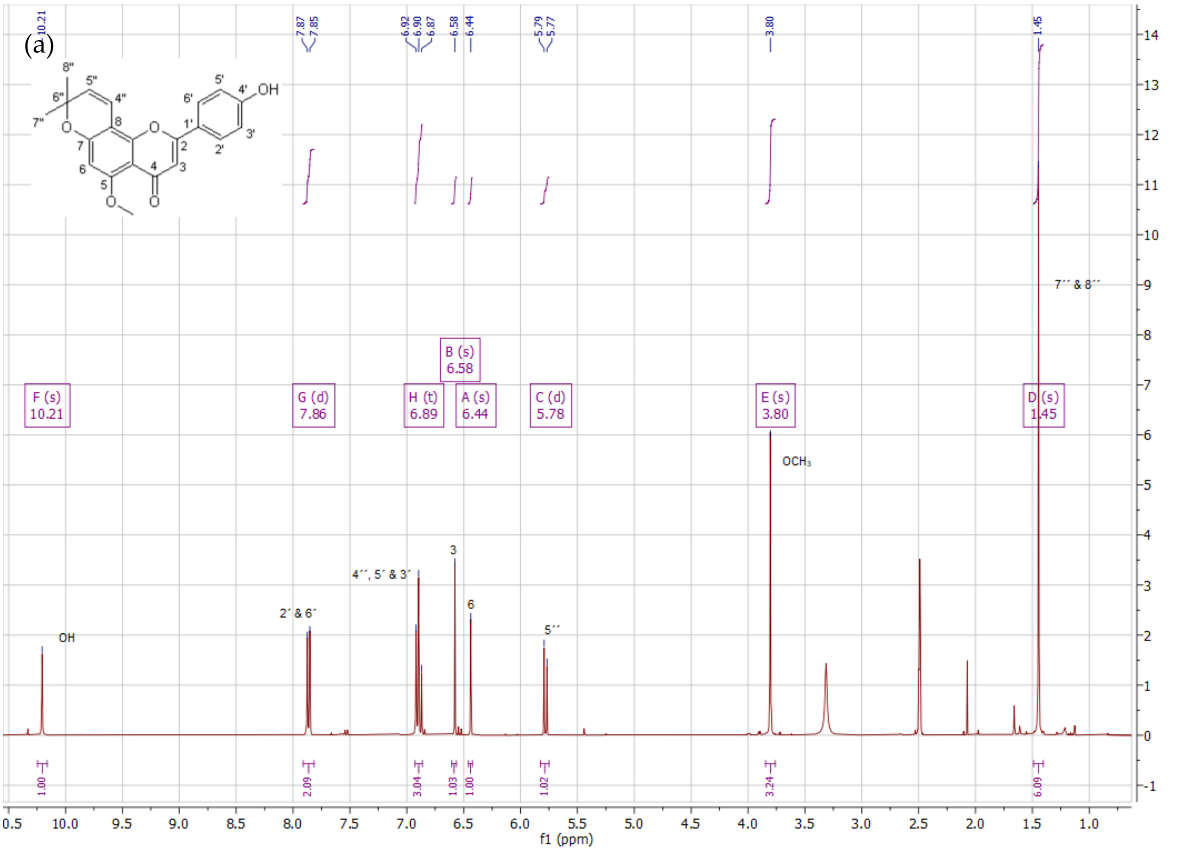

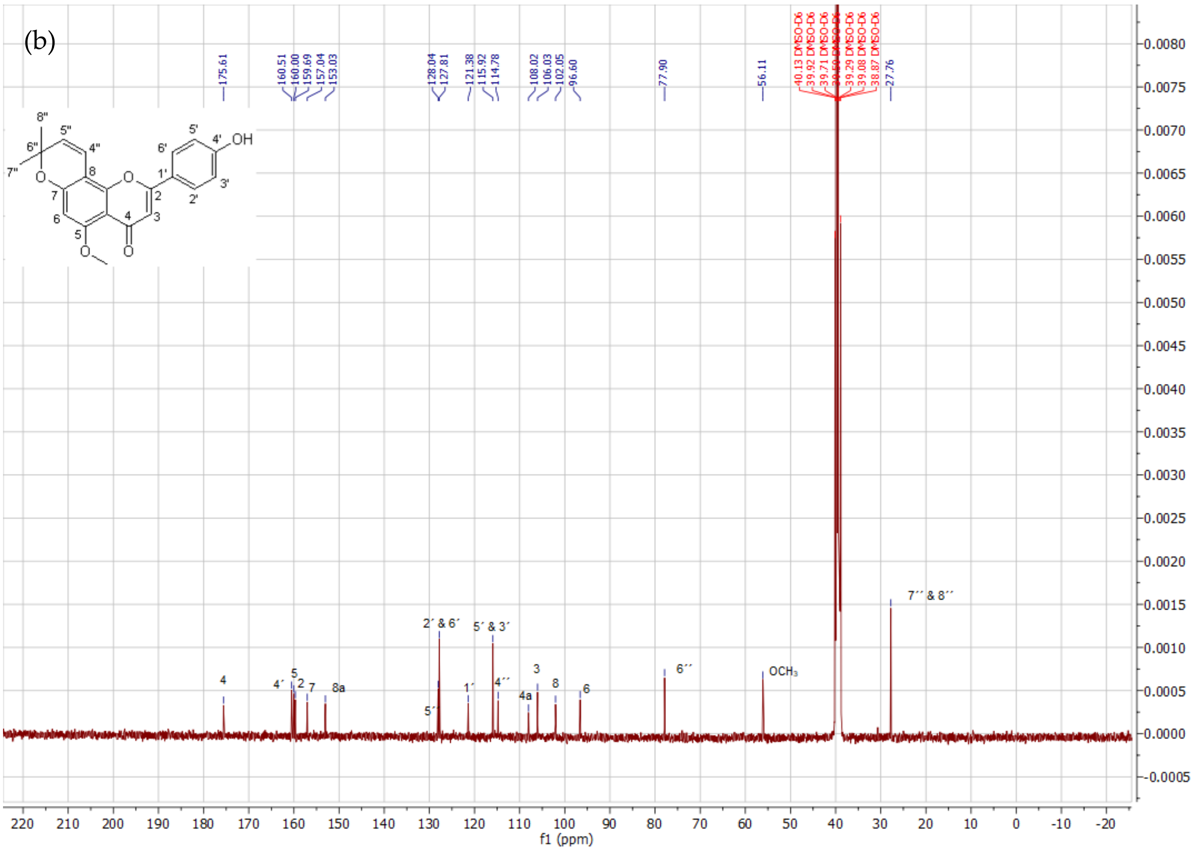

(c)

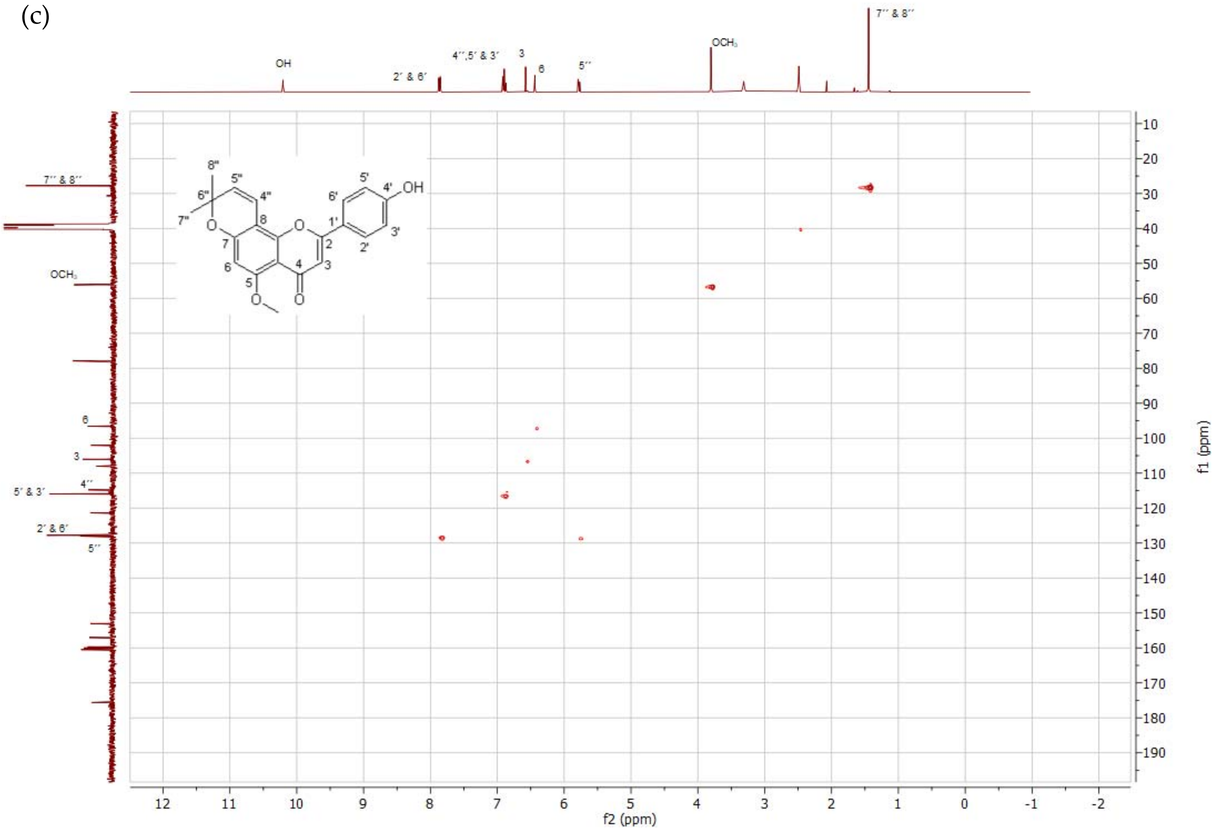

(d)

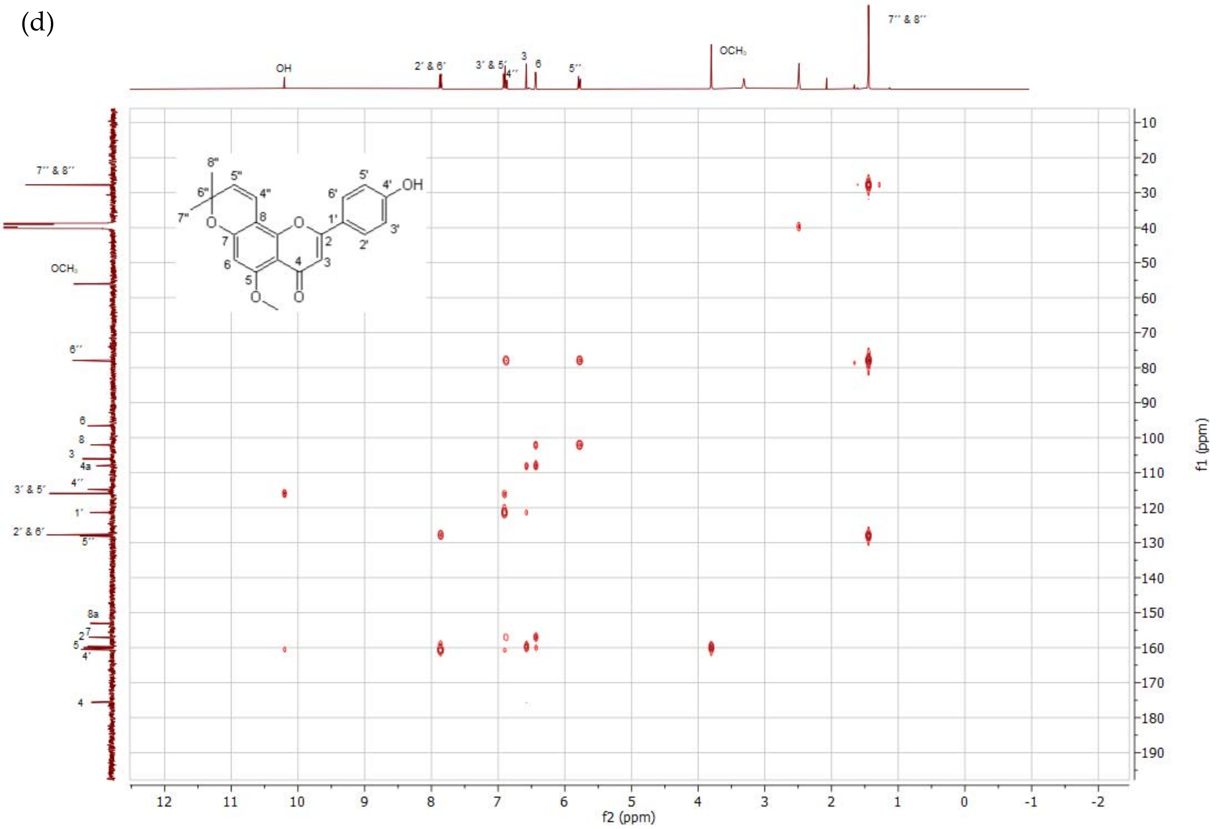

Figure S3. (**a**) ^{1}H-NMR, (**b**) ^{13}C-NMR, (**c**) HSQC and (**d**) HMBC of pyranoflavone (**5**).

4. NMR spectra of pyranoaurone (6)

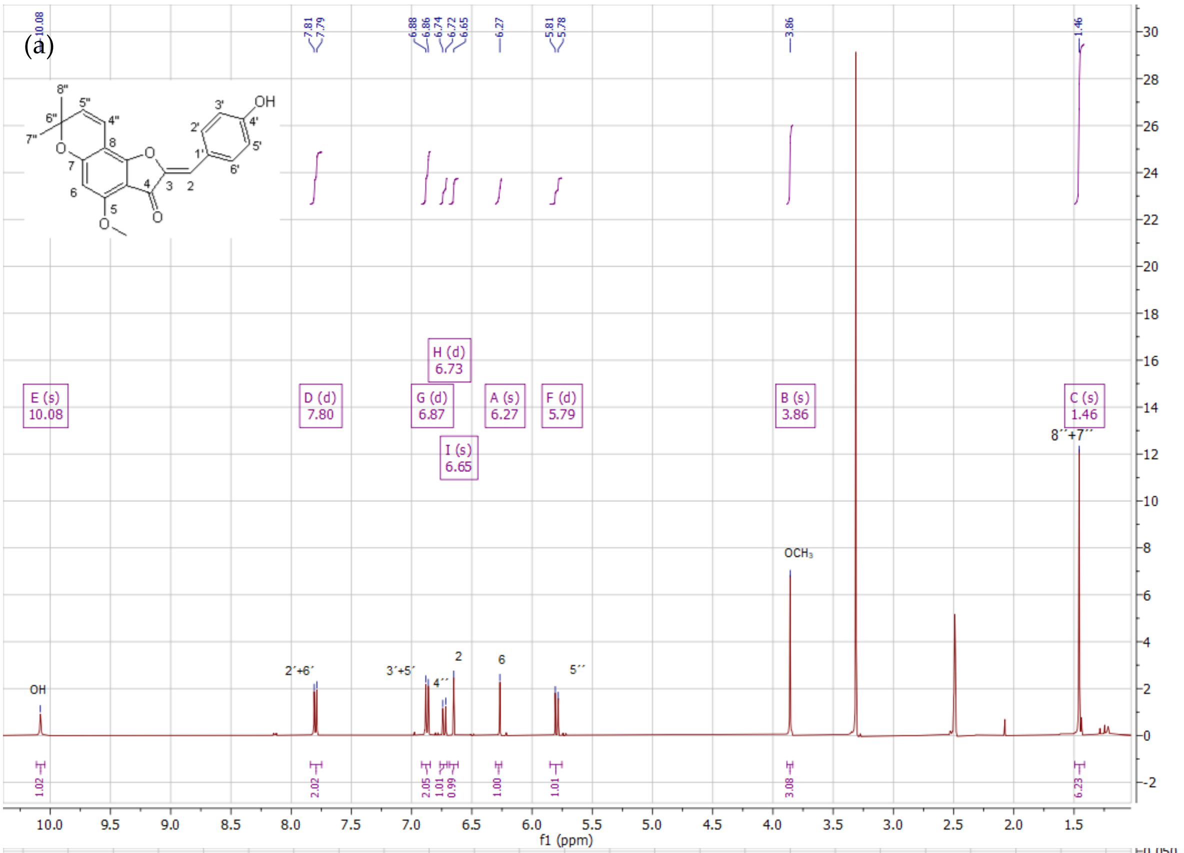

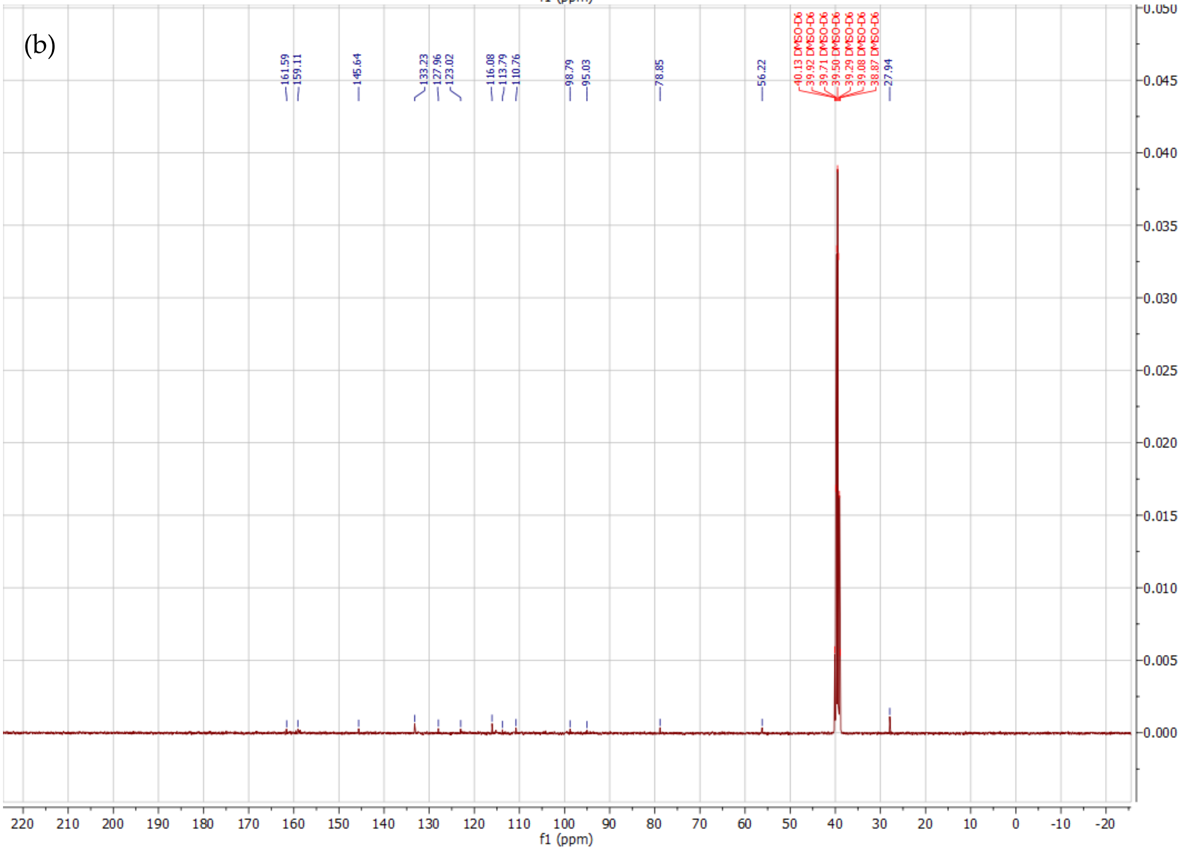

(c)

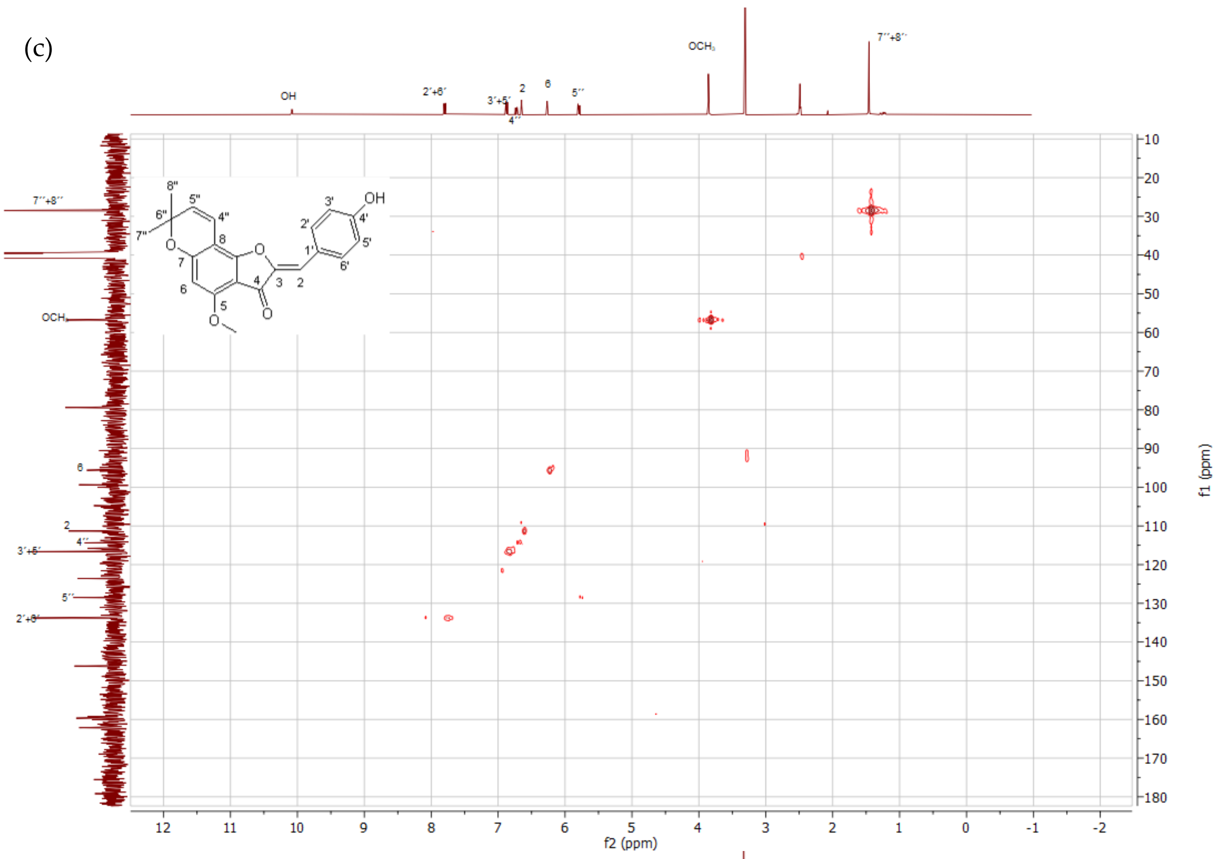

(d)

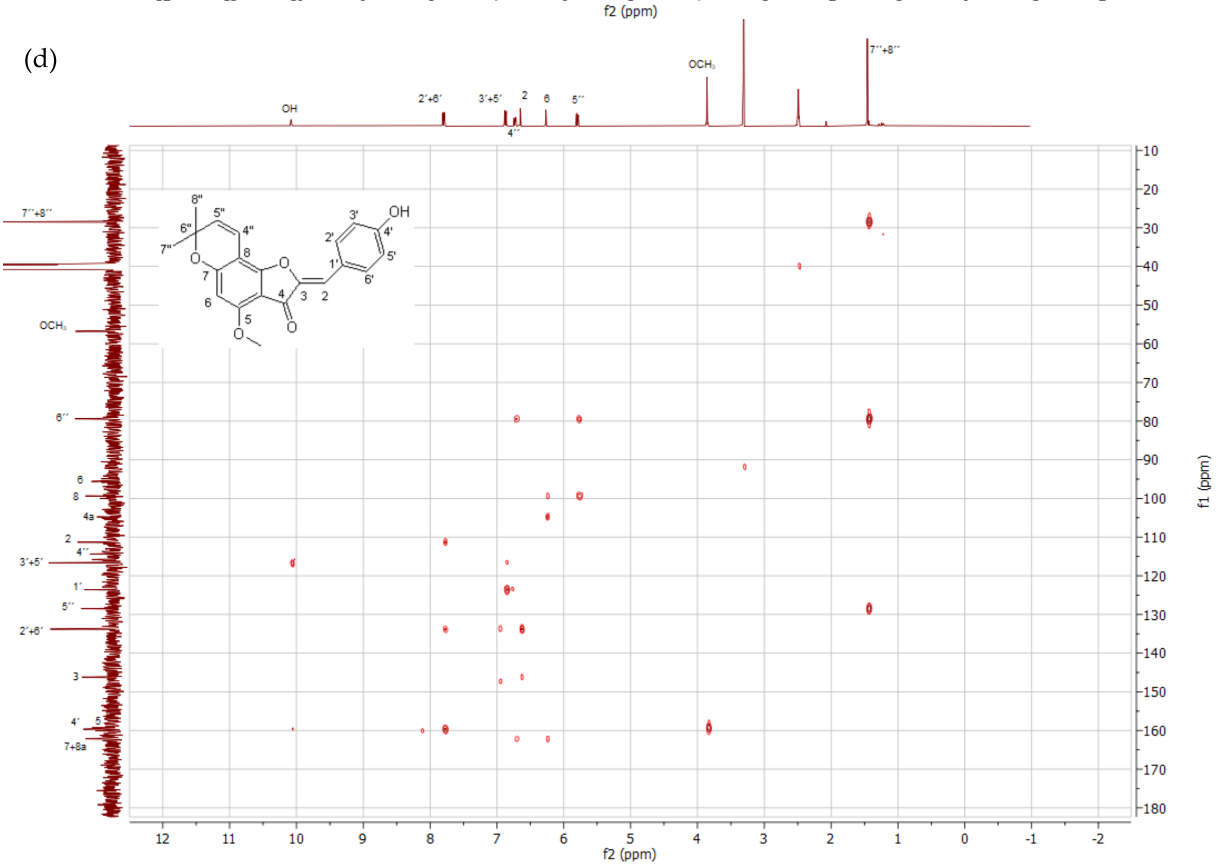

Figure S4. (**a**) ^{1}H-NMR, (**b**) ^{13}C-NMR, (**c**) HSQC and (**d**) HMBC of pyranoaurone (**6**).

5. NMR spectra of pyranoflavonol (7)

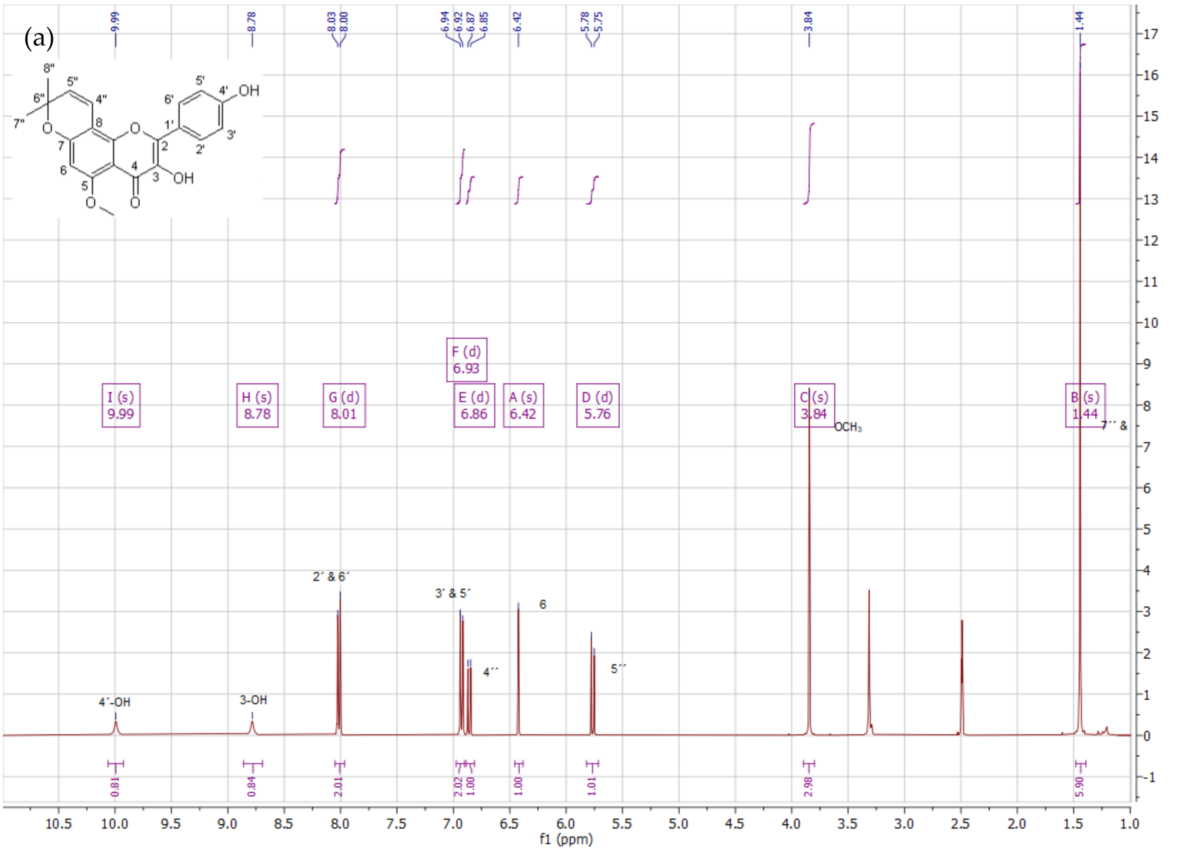

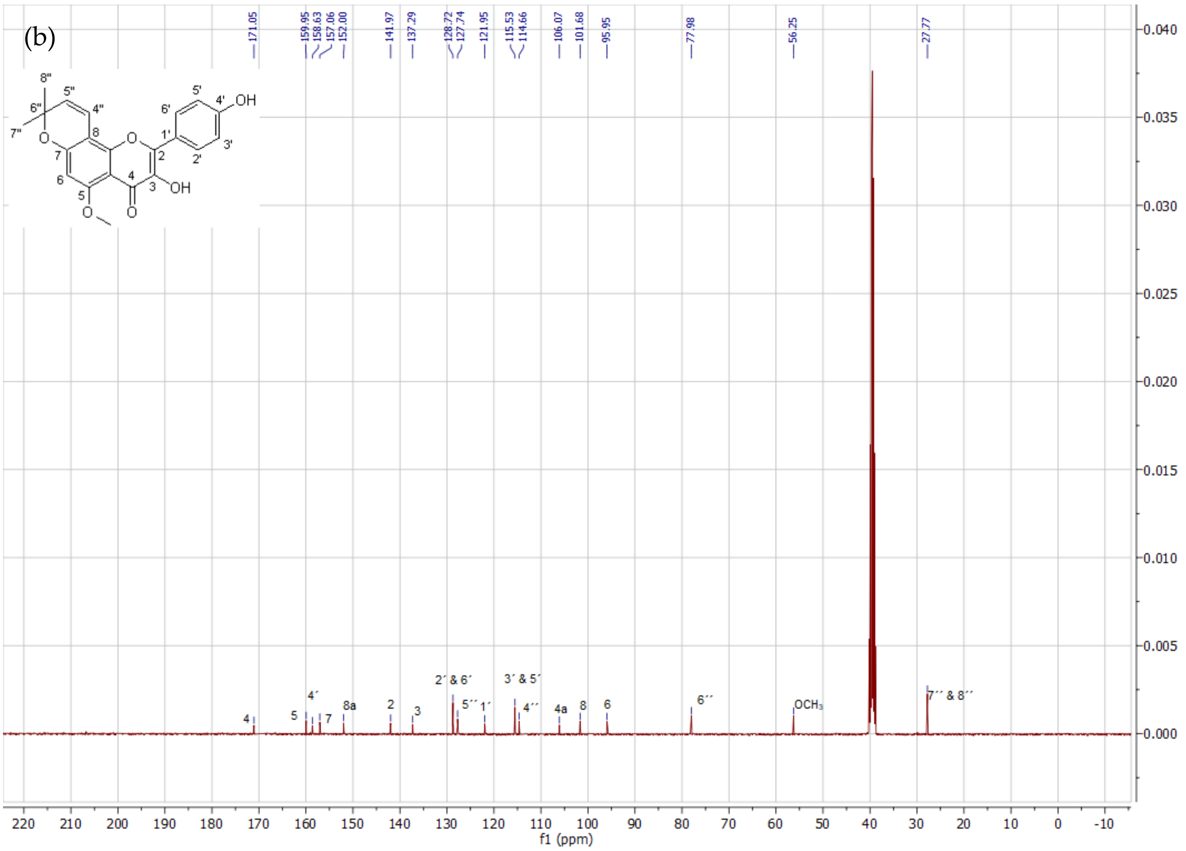

(c)

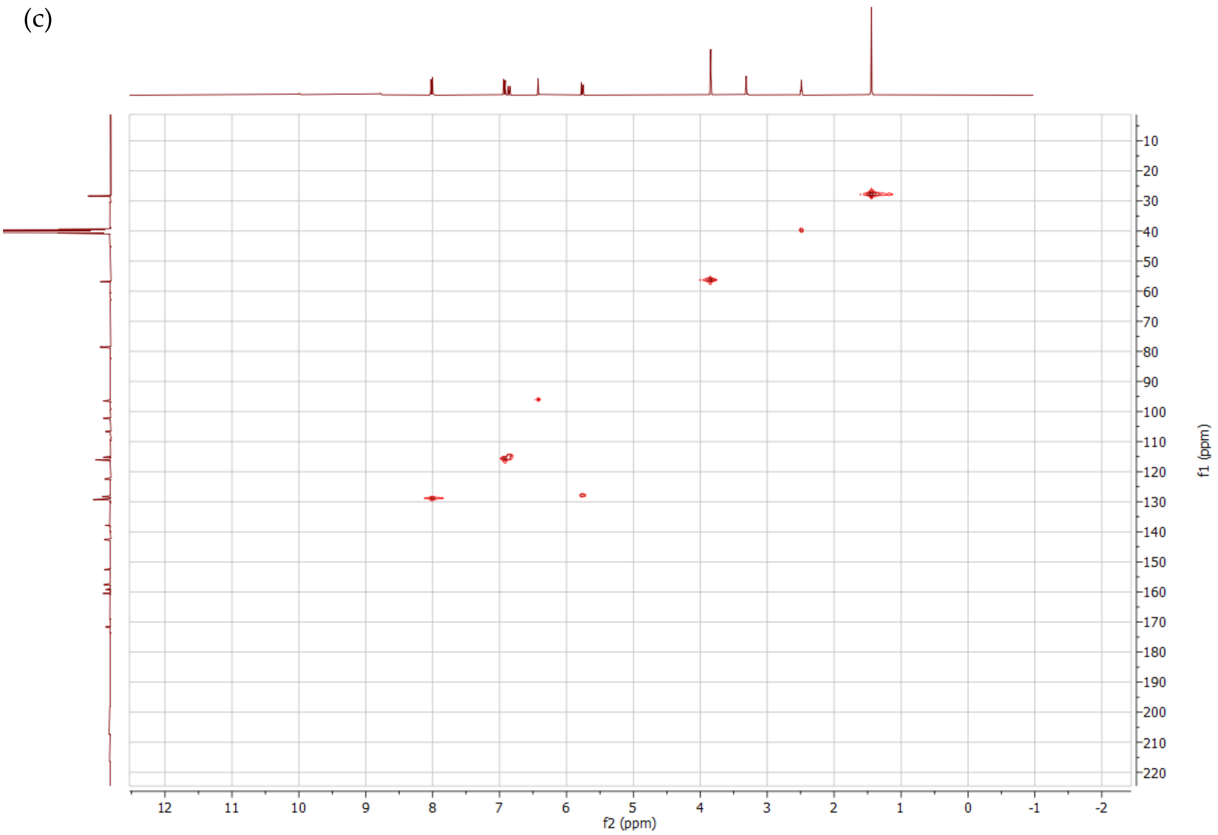

(d)

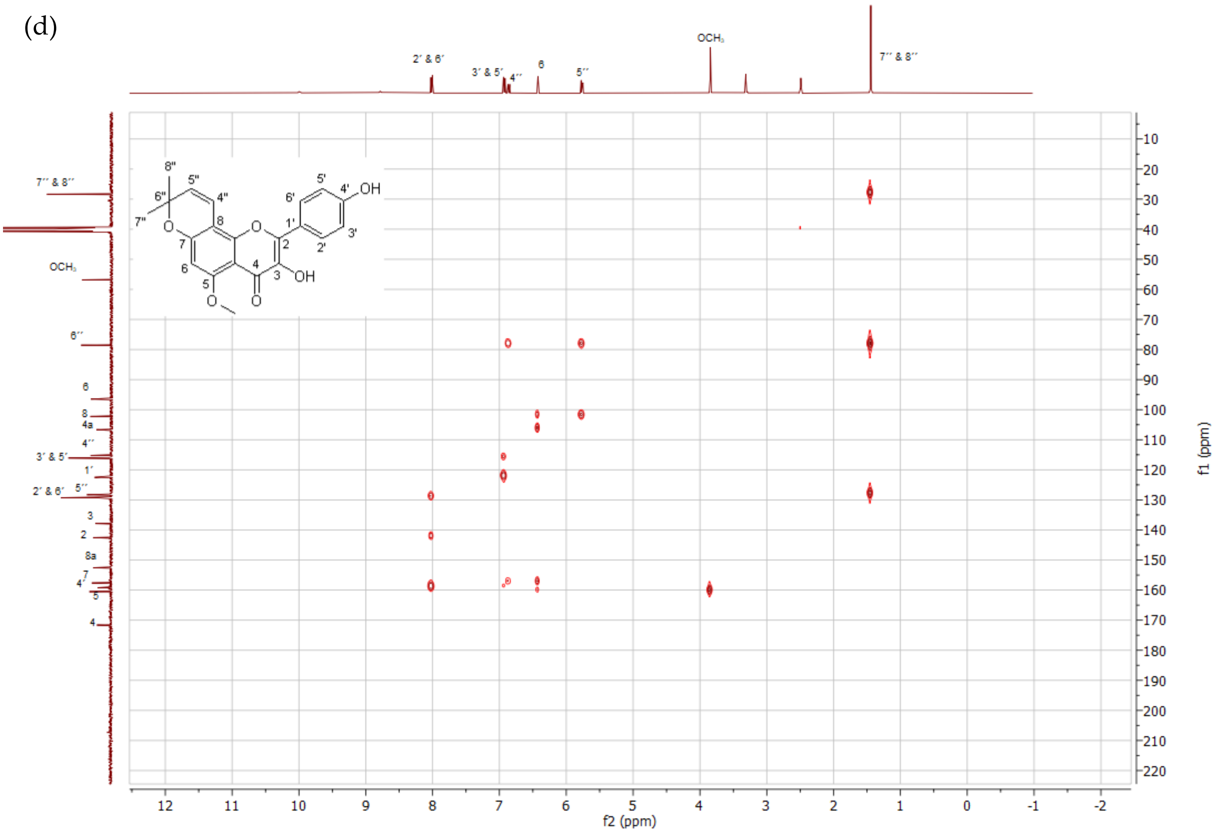

Figure S5. (**a**) ^{1}H-NMR, (**b**) ^{13}C-NMR, (**c**) HSQC and (**d**) HMBC of pyranoflavonol (**7**).
